# Using Event Related Potentials to Characterise Inhibitory Control and Self-Monitoring Across Impulsive and Compulsive Phenotypes: A Dimensional Approach to OCD

**DOI:** 10.1017/S109285292200075X

**Published:** 2022-04-29

**Authors:** Sakshi Dhir, Kaelasha Tyler, Lucy Albertella, Samuel R. Chamberlain, Wei-Peng Teo, Murat Yücel, Rebecca A. Segrave

**Affiliations:** 1 BrainPark, Turner Institute for Brain and Mental Health, School of Psychological Sciences and Monash Biomedical Imaging Facility, Monash University, Melbourne, Victoria, Australia; 2 Department of Psychiatry, Faculty of Medicine, University of Southampton, UK; & Southern Health NHS Foundation Trust, UK; 3 Physical Education and Sports Science Academic Group, National Institute of Education, Nanyang Technological University, Singapore; 4 Institute for Physical Activity and Nutrition, Deakin University, Melbourne, Australia

## Abstract

**Objective:**

‘Subsyndromal’ Obsessive Compulsive Disorder symptoms (OCDS) are common and cause impaired psychosocial functioning. OCDS are better captured by dimensional models of psychopathology, as opposed to categorical diagnoses. However, such dimensional approaches require a deep understanding of the underlying neurocognitive drivers and impulsive and compulsive traits (i.e., neurocognitive phenotypes) across symptoms. This study investigated inhibitory control and self-monitoring across impulsivity, compulsivity and their interaction in individuals (*n* = 40) experiencing mild-moderate OCDS.

**Methods:**

EEG recording concurrent with the stop signal task was used to elicit event related potentials (ERPs) indexing inhibitory control (i.e., N2 and P3) and self-monitoring (i.e., ERN and CRN: negativity following erroneous or correct responses, respectively).

**Results:**

During unsuccessful stopping, individuals high in both impulsivity and compulsivity displayed enhanced N2 amplitude, indicative of conflict between the urge to respond and need to stop (*F* (3, 33) = 1.48, *p* < .05, 95% CI [-.01, .001]). Individuals high in compulsivity and low in impulsivity showed reduced P3 amplitude, consistent with impairments in monitoring failed inhibitory control (*F* (3, 24) = 2.033, *p* < .05, 95% CI [-.002, .045]). Following successful stopping, high compulsivity (independent of impulsivity) was associated with lower CRN amplitude, reflecting hypo-monitoring of correct responses (*F* (4, 32) = 4.76, *p* < .05, 95% CI [.01, .02]), and with greater OCDS severity (*F* (3, 36) = 3.32, *p* < .05, 95% CI [.03, .19]).

**Conclusion:**

The current findings provide evidence for differential, ERP indexed inhibitory control and self-monitoring profiles across impulsive and compulsive phenotypes in OCDS.

## Introduction

Ritualistic behaviours (i.e., compulsions) in response to intrusive thoughts and/or images (i.e., obsessions) impact approximately 25% of people at some point in their life ^1–4^. These highly prevalent OCD symptoms (OCDS), that is symptoms regardless of whether they meet a diagnostic threshold, are associated with disruptions in psychosocial functioning and psychological distress ^2^. For instance, there is compelling evidence from a large data set (n = 7076) that people experiencing subsyndromal symptoms and those with OCD, as compared to those with no lifetime experience of obsessive thoughts and compulsive behaviours, show similar impairments in physical health, functioning, psychological vulnerabilities and psychiatric co-morbidities^5^. Further, there is evidence of dimensional OCDS being associated with worsened quality of life across work, relationships and leisure when controlling for other variables^6^. In addition, it has been documented that approximately 1.2% of people who experience OCDS go on to develop Obsessive Compulsive Disorder (OCD), and OCDS are common across other mental illnesses, such as anxiety disorders, and impulse control disorders such as Tourette Syndrome and substance use disorders ^1,2,7^. The categorical diagnostic approach in psychiatric classification systems (DSM-5 and ICD-11), however, do not recognise subsyndromal symptoms ^8^. An alternative and increasingly utilised means of understanding ‘sub-clinical’ psychopathology, such as OCDS, is to take a dimensional approach and investigate the traits and neurocognitive drivers, i.e., neurocognitive phenotypes, that underpin a large breadth of these symptoms^9,10^. This approach i) enables identification of subsyndromal presentations, and ii) promotes prevention and early intervention approaches targeting underlying drivers, as opposed to attempting treatment only when symptoms are more ingrained and meet diagnostic thresholds.

Impulsivity and compulsivity are two interlinked dimensional traits core to OCDS. Impulsivity is a tendency towards strong urges without forethought, often associated with short-term reward ^11–13^, whereas compulsivity is a tendency towards repetitive behaviours accompanied by the feeling that one ‘has to’ perform them, coupled with an awareness that they are incongruent with overall goals ^14^. Whilst both traits result in the common outcome of dyscontrol over behaviour, they are classically held to be driven by differing underlying motivations: impulsivity by an urge to obtain reward and compulsivity by the desire to avoid harm, fear of uncertainty and/or habit ^15–20^. Traditionally impulsivity and compulsivity were considered to be orthogonally opposed, however recent evidence show a more complex interdependent relationship where, particularly at the extreme ends, they interact in a way that is reflected in specific clinical outcomes ^21–23^. These different loadings of impulsivity and compulsivity are reflected as distinct phenotypes. People high in both traits have shown more chronic ^24^ and severe OCDS ^25^ with poorer prognosis ^26^. Thus, characterising the overlapping and distinct underlying drivers of impulsivity, compulsivity and their interaction in OCDS may uncover particularly ‘at risk’ individuals, facilitating intervention before symptoms escalate.

Two key neurocognitive drivers of impulsivity and compulsivity that may hold the key to better understanding OCDS are inhibitory dyscontrol and impaired self-monitoring. Inhibitory dyscontrol refers to the inability to withhold a response, such as difficulty resisting compulsive urges to wash hands, and is common to both impulsivity and compulsivity ^27–31^. Hyperactivity self-monitoring, which has been robustly implicated in high compulsivity ^27,32–34^, involves persistent observing, checking and questioning ‘correct’ performance, which is often described as the feeling of something being ‘not just right’. This doubt about performance being ‘correct’, for example doubt that hands are clean, triggers a compensatory system in the form of compulsive behaviours such as excessive handwashing. On the other hand, hypoactive self-monitoring has been shown, albeit with limited evidence, in high impulsivity ^35^. Such nuanced commonalties in inhibitory control and differences in self-monitoring between compulsivity and impulsivity may distinctly drive OCDS. For example, a person with high impulsivity could be characterised by impaired inhibitory control and hypoactive self-monitoring, and as such experience strong impulses to engage in repetitive handwashing. Whereas a person with high compulsivity could experience the same handwashing behaviour, but be driven by impaired inhibitory control and hyperactive self-monitoring, and associated doubt about the cleanliness of their hands ^36^. Thus, characterising inhibitory control and self-monitoring across impulsive and compulsive phenotypes may identify the unique individual’s drivers of OCDS.

**Figure 1:**
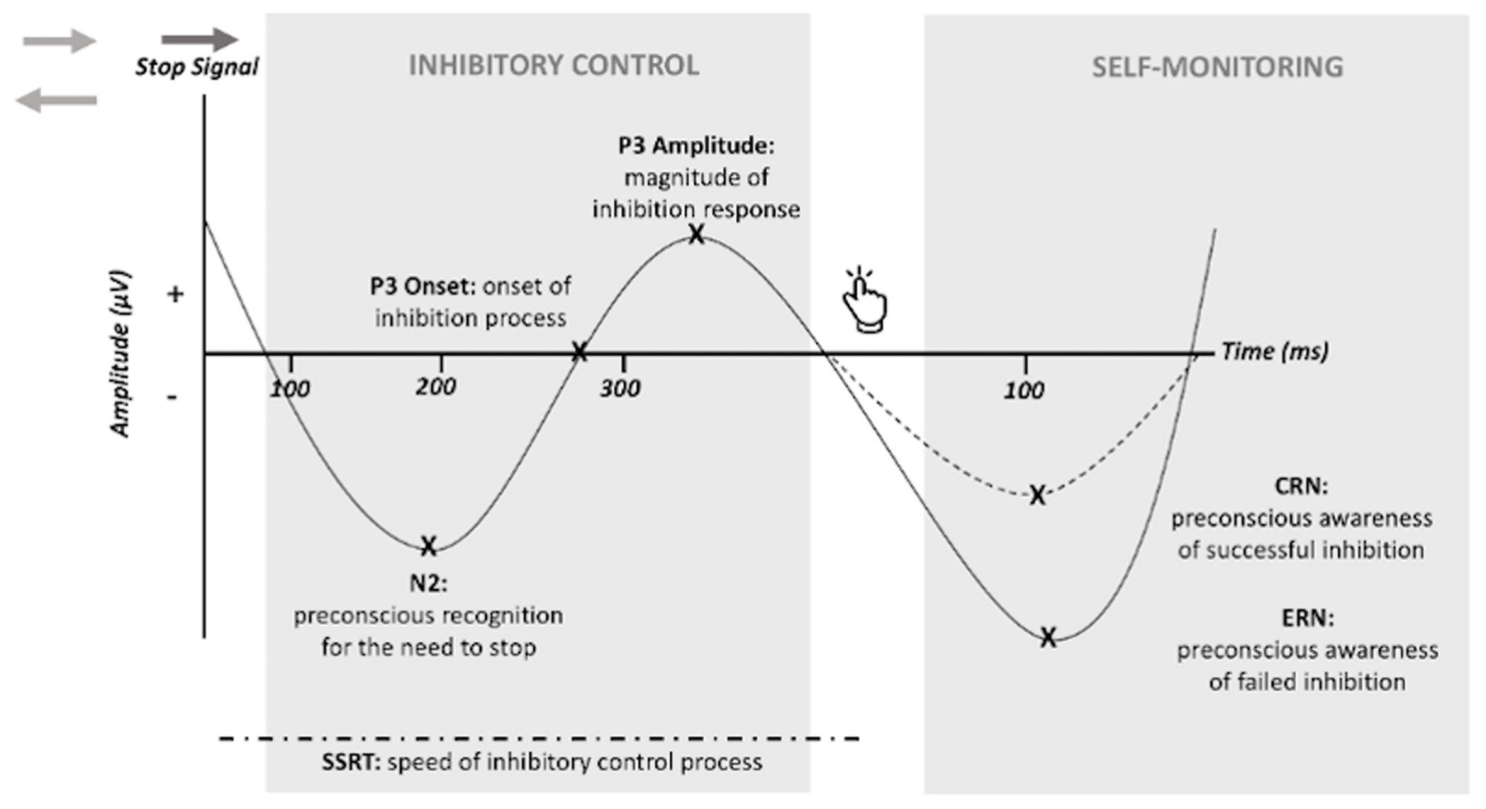
Schematic Representation of N2, P3 and ERN/CRN during The Stop Signal Task Abbreviation: Ms, Milliseconds. μV, Microvolts. SSRT, Stop Signal Reaction Time. Note. During the Stop Signal Task, participants are required to respond via button press (left or right arrow) to a Go signal (grey arrows), however in some cases the Go signal is followed by a ‘Stop Signal’ (red arrow) and they are required withhold the initiated urge to respond. The SSRT measures the time from the stop signal to the inhibition response, thus indicates inhibition speed. Discrete neurocognitive components of the inhibitory control process are reflected in ERPs, as follow: N2 amplitude = pre-conscious awareness of the need to stop, P3 onset latency = onset of the inhibition process, P3 amplitude = the magnitude of inhibition response, CRN = monitoring of successful inhibition, and ERN = monitoring of failed inhibition.

Event related potentials (ERPs) are time-locked electrophysiological brain responses elicited in direct response to sensory, cognitive, or motor events. A number of ERPs have been directly linked to specific neurocognitive aspects of inhibitory control and self-monitoring. Inhibitory control is strongly associated with the N2 (a negative ERP deflection approximately 200ms after encountering a cue to ‘stop’ a response), with a greater N2 amplitude thought to reflect the strength of one’s preconscious recognition of the need to stop ^37,38^. There is also a large body of evidence indicating that the latency of the P3 (a positive ERP deflection approximately 300ms after the stop signal) is a sensitive index of the onset of the inhibition process ^39^ and the amplitude reflects the magnitude of the inhibition response ^40–42^. Moreover, the strength of self-monitoring has been strongly associated with the magnitude of Error-Related Negativity (ERN) and Correct-Related Negativity (CRN) (negative deflections 100ms after failed (i.e., ‘an error’) or successful inhibition, respectively)^33^ (Figure 1). Thus, these ERPs provide a highly sensitive means of investigating the common and distinct neurophysiological mechanisms of inhibitory control and self-monitoring across impulsivity and compulsivity, and as such uncovering the neurocognitive phenotypes across OCDS.

The evidence for N2/P3 and ERN/CRN across impulsivity and compulsivity is sparse. There is evidence^43^ of an enhanced N2 amplitude in OCD, an archetypal compulsive condition, compared to gambling disorder, an archetypal impulsive condition. The authors positioned the early inhibitory control process, indexed by the N2, as a candidate *differential* phenotype for compulsivity as compared to impulsivity. Additionally, a comprehensive systematic review^44^ of ERPs associated with OCD provided evidence of enhanced ERN, and associated hyperactive self-monitoring, as an endophenotype for OCD. These finding highlighted the utility of ERPs for neurocognitively phenotyping OCDS. However, the scant research that has been conducted in this area has focused exclusively on inhibitory control and self-monitoring related ERPs in OCD, rather than investigating whether they are sensitive markers of impulsivity and compulsivity dimensionally. Thus, the extent to which inhibitory control, indexed by N2/P3, and self-monitoring, indexed by ERN/CRN, are makers across impulsivity and compulsivity in OCDS remains unknown.

In sum, impulsivity and compulsivity play an important, complex, differential, and only partially understood role in driving OCDS. The identification of impulsive and compulsive neurocognitive phenotypes across OCDS would contribute to early detection and targeted early intervention efforts. Impairments in inhibitory control and self-monitoring are central to impulsivity and compulsivity and can be sensitively indexed via ERPs. Thus, the current study attempted to neurocognitively phenotype impulsivity and compulsivity in OCDS by investigating the extent to which impulsivity, compulsivity, and their interactions were associated with impairments in inhibitory control indexed by N2 and P3, and self-monitoring indexed by ERN and CRN, in people with mild to moderate OCDS. It was hypothesised that greater compulsivity would be associated with hyper self-monitoring, reflected by enhanced ERN/CRN, and greater impulsivity with hypo self-monitoring, reflected by reduced ERN/CRN. Additionally, it was hypothesised that high impulsivity and compulsivity would both be associated with a range of inhibitory control impairments, reflected by altered N2 and P3.

## Method

### Participants

Forty right-handed adults (female = 33, male = 7; M±SD years = 24.25 ± 5.20) experiencing mild (*n =* 16) to moderate (*n =* 24) OCDS took part in the study, with mild severity defined by scores between 8-15 and moderate by scores between 16-23 on the Yale Brown Obsessive Compulsive Scale-Revised (YBOCS-R) ^45^. All participants had normal or corrected-to-normal vision and met the exclusion criteria of no lifetime history of DSM-5 defined psychotic illness, Bipolar Affective Disorder, Bulimia or Anorexia Nervosa, Substance Use or Gambling Disorder, neurological illness or moderate - severe brain injury and stimulant medication use. Participants were recruited through social media, posters placed around the community, and an anxiety and OCD helpline. The study received approval from the Monash University Human Ethics Committee and all participants provided informed consent. Participants were paid $40 to compensate for their time and effort.

### Procedure

Data were collected during a single experimental session conducted at the Monash Biomedical Imaging Centre and BrainPark, Melbourne. The experimental protocol began with a clinical interview and questionnaires to collect demographic, diagnostic and psychological data. All participants completed the Stop Signal Task (SST) with concurrent EEG recording. All clinical interviews, questionnaires and cognitive tasks were administered by a single researcher who was a provisional psychologist and trained in their standardised administration. See Supplementary Material Section A for a detailed outline of all measures.

### Interviews

OCDS severity was assessed by the Yale Brown Obsessive Compulsive Scale-Revised (YBOCS-R) ^45^. The YBOCS-R is a gold-standard frequently utilised measure of obsession and compulsion symptom severity ^10^.

Exclusion criteria was assessed by the Mini-International Neuropsychiatric Interview (MINI) ^46^. The MINI is a diagnostic research tool used to assess whether a person meets criteria for current or past common DSM-IV defined mental illnesses.

### Questionnaires

The use of the following self-report questionnaires to capture dimensional impulsivity and compulsivity has been applied across a range of prior studies, and reliably differentiates between the two traits ^47–49^. Compulsivity was assessed by the composite total score of The Obsessional Beliefs Questionnaire ^50^ a 44-item scale used to measure the level of obsessional beliefs, and The Intolerance of Uncertainty Scale ^51^, a 12-item scale used to assess responses to uncertain and ambiguous possibilities, including the future. Impulsivity was assessed by the total score on the short version of the Urgency, Premeditation, Perseverance, Sensation Seeking and Positive Urgency, Impulsive Behaviour (UPPS-P), a 59 item scale used to measure domains of impulsivity ^52^.

The Warwick Edinburgh Mental Well-being Scale (WEMWBS)^53^, a 14-item scale, was used to measure overall wellbeing and psychological functioning. Higher scores indicate a higher level of wellbeing. The WEMWBS shows high levels of internal consistency, reliability and usefulness at a population-level. A WEMWM score <40 has been found across populations accessing secondary care mental health services^54^.

### Stop Signal Task

Inhibitory control was assessed using a gold-standard version of the Stop-Signal task (SST) which had been shown to sensitively elicit inhibitory control related ERPs ^39,55^. The SST was run via MATLAB (version 2019b). A fixation cross was presented on the monitor screen for 500ms, followed by the target ‘Go’ stimuli indicated by a white arrow, which required a response with the corresponding left/right computer key (response deadline/presentation: 1000ms). On 33% of the trials, the ‘Go’ stimuli was followed by the ‘Stop’ stimuli, indicated by a red arrow (presented for 100m), which required participants to withhold their response. The longer the stop signal delay (SSD; the time between the ‘Go’ and ‘Stop’ stimuli), the more difficult it is for participants to successful stop a response. The SSD was adjusted by 50ms increments (starting at 200ms) to ensure adaptive difficulty and an accuracy rate of 50% for all participants. The behavioural metric collected was the Stop Signal Reaction Time (SSRT), which is the time required for a person to stop a response. The SSRT was calculated based on the integration method ^56^.

Participants completed a total of 240 trials presented in two equal blocks with a short break between blocks. Prior to beginning the task, participants completed a practice trial. The instructions for the task were standardised across all participants (“respond as fast as possible, whilst trying to maintain accuracy”), and a researcher was present throughout task completion to monitor engagement. If participants were observed to strategically slow responses, they were instructed to “remember to respond as fast as possible”.

### Electrophysiological Recording and Pre-Processing

EEG was recorded in a darkened and electrically shielded room using a digital Active-Two system (BioSemi, Amsterdam, Netherlands). Silver chloride (Ag/AgCl) active electrodes were placed at 10 scalp sites (*F3, Fz, F4, C3, Cz, C4, P3, Pz, P4, AFz*) according to the international 10-20 montage system. Four facial electrodes were positioned adjacent to the left and right outer canthus of each eye and above and below the left orbit to measure eye movement. EEG data were referenced to mastoid channels and impedances were kept below 5KOhms. Key presses were detected using a regular PC keyboard, which fed triggers to the Active-Two system via the PC serial port. All signals were digitized with a sampling rate of 1000 Hz, a 24-bit A/D conversion and a low pass filter of 134.

Offline data were processed with EEG LAB open-source toolbox ^51^. EEG data were re-referenced to a common average and the mastoid channels removed from further analysis. Data were down sampled to 500 Hz and further filtered using a linear basic FIR filter with a high edge of the frequency band pass of 40 Hz and a low edge of the frequency band pass of 1 Hz. The data were then epoched from 500 ms prior to a Go stimulus to 1500 ms after the Go stimulus. Epochs containing motion artefact were removed. The EEG data set for each participant was then subject to a temporal Independent Component Analysis (ICA) decomposition using the runica infomax algorithm. Based on visual inspection, any components relating clearly to electro-oculagram (EOG) or eye blink artifacts were excluded. EOG channels were then removed from further analysis.

All baseline means (i.e., the average microvolt value from -100 ms to 0 ms relative to the Go signal) were removed across trials. Responses made within 50ms of all stimuli presentations were considered as early responses and omitted from analysis. Trials were grouped into: (1) Successful stop trials (trials where participants did not respond after the ‘stop signal’), (2) Failed stop trials (trials where participants incorrectly responded after the ‘stop signal’) and (3) Go trials (trials where participants were not required to inhibit a response). Stop trials with SSDs < 50ms were excluded to safeguard the adaptive difficulty process. Go and stop trials were matched based on SSD to ensure homogenous parameters for comparison of ERPs. Data were re-epoched at -100ms to 500 ms relative to stop signal onset. Latency or amplitude values for P3, N2, ERN and CRN were calculated at the fronto-central EEG channels Cz and Fz. However, consistent with the literature, P3 values reported were those calculated at Fz, and N2, ERN and CRN values reported were those calculated at Cz ^39,58–60^

### EEG: Inhibitory Control ERP Calculation

N2 and P3 ERPs were used to index underlying inhibitory control processes. The N2 amplitude was defined as the most negative value within the 200–300ms interval post-stimulus onset, and the P3 amplitude as the most positive value within the 300–500ms interval. A modified version of the COMPASS algorithm^39^ was used to increase signal to noise ratio of N2 and P3 waveforms and to remove ICA derived components which did not represent the N2 and P3 from the analysis (see Supplementary Material Section B). This allowed selection only of those ICA derived components that represented the N2 across successful (*n*= 37) and failed (*n*= 37) stop trials. Similarly, components were selected that represented P3 onsets within successful (*n*= 28) and failed (*n*= 30) stop trials, and P3 amplitudes across successful (*n* =28) and failed (*n* =28) stop trials.

### EEG: Self-Monitoring ERP Calculation

ERN and CRN were used to index underlying self-monitoring. ERN was defined as the average peak (*μ*V) from 0-100ms after failed stop trials, and Correct-Related Negativity (CRN), as the average peak (*μ*V) after successful stop trials. Pre-processed data were re-epoched from -400 ms to 800 ms around participant responses. Baseline means (i.e., the average microvolt value) were calculated from 400ms to 200ms prior to participant response. The baseline averages were then removed all across trials^61^. This allowed the selection only of components representing ERN (n= 37) and CRN (n= 37).

### Statistical Analysis

All statistical analyses were conducted on SPSS and PROCESS. Outliers (*n* = 5 across ERP data) were winsorised (based on Z scores > 3.29). Firstly, to determine differences associated with successful/failed inhibitory control outcomes, independent sample tests were used to analyse group differences between (1) successful and failed stop trials across N2 amplitude and P3 onset and amplitude and (2) ERN and CRN. Then, bootstrapped linear regressions examined whether impulsivity, compulsivity, or their interaction were associated with (1) cognitive inhibitory control outcomes (SSRTs), (2) symptom severity (YBOCS scores), and (3) ERP indices of inhibitory control (N2 Amplitude, P3 Onset, and P3 Amplitude) and self-monitoring (ERN and CRN) across failed and successful stop trials. Impulsivity and compulsivity scores were mean centred according to the respective outcome group, and interaction terms were calculated accordingly to avoid multicollinearity. The unstandardised beta (B) has been reported for all significant (*p* < .05) main effects, i.e., the amount of change in the outcome associated with every unit change in the predictor. Interaction effects were valid given that the independent variables (impulsivity and compulsivity, as operationalised above) were not correlated (*p* = .77). Significant interaction effects were followed up by (1) splitting the groups by impulsivity, and then within each group conducting correlations between compulsivity and the outcome measure and (2) assessing scatterplots to facilitate interpretation^62^. Age, gender and anxiety were controlled in the model when they significantly correlated with the dependent variables, which was only evident for age and CRN.

## Results

### Participant Characteristics

Participants (*n* = 40) were all within the mild (YBOCS-R score = 8 - 15) to moderate (YBOCS-R score = 16 – 23) range of OCDS. The MINI clinical interview and DSM-5 criteria indicated that 15 participants met criteria for OCD, and 25 were experiencing subclinical OCDS. The WEMWBS wellbeing scores were low for participants with mild and moderate symptoms, as compared to norms for healthy young adults^53^ and were comparable to the norms for people accessing secondary care mental health services^54^ (see Table 1). Participants showed longer SSRTs than the normative mean for healthy young people ^*63*^, showed more delay P3 onset latencies than previously found using the same SST^*39*^ and showed more negative ERN and CRNs compared to the normative data recorded at Cz^*61*^ (see Table 1).

### OCDS Severity and Stop Signal Reaction Time

#### OCDS Severity

Collectively, impulsivity, compulsivity, their interaction accounted for 46.5% of variance in YBOCS scores (*R^2^* = .465). Higher compulsivity was associated with significantly greater YBOCS scores, (F (3, 36) = 3.32, *p* < .05, 95% CI [.03, .19]), such that for every unit increase in compulsivity, YBOCS scores increased by .09 units (*B* = 0.09 (*SE* = .03)). Neither impulsivity or the interaction between impulsivity and compulsivity were associated with variation in YBOCS scores (*p*’s > .31).

#### Stop Signal Reaction Time

Impulsivity, compulsivity, and their interaction were not associated with significant variation in SSRT (*F* (3, 36) = 1.998, *p* = .13).

### Successful versus Failed Stopping Across all ERPs

**Figure 2:**
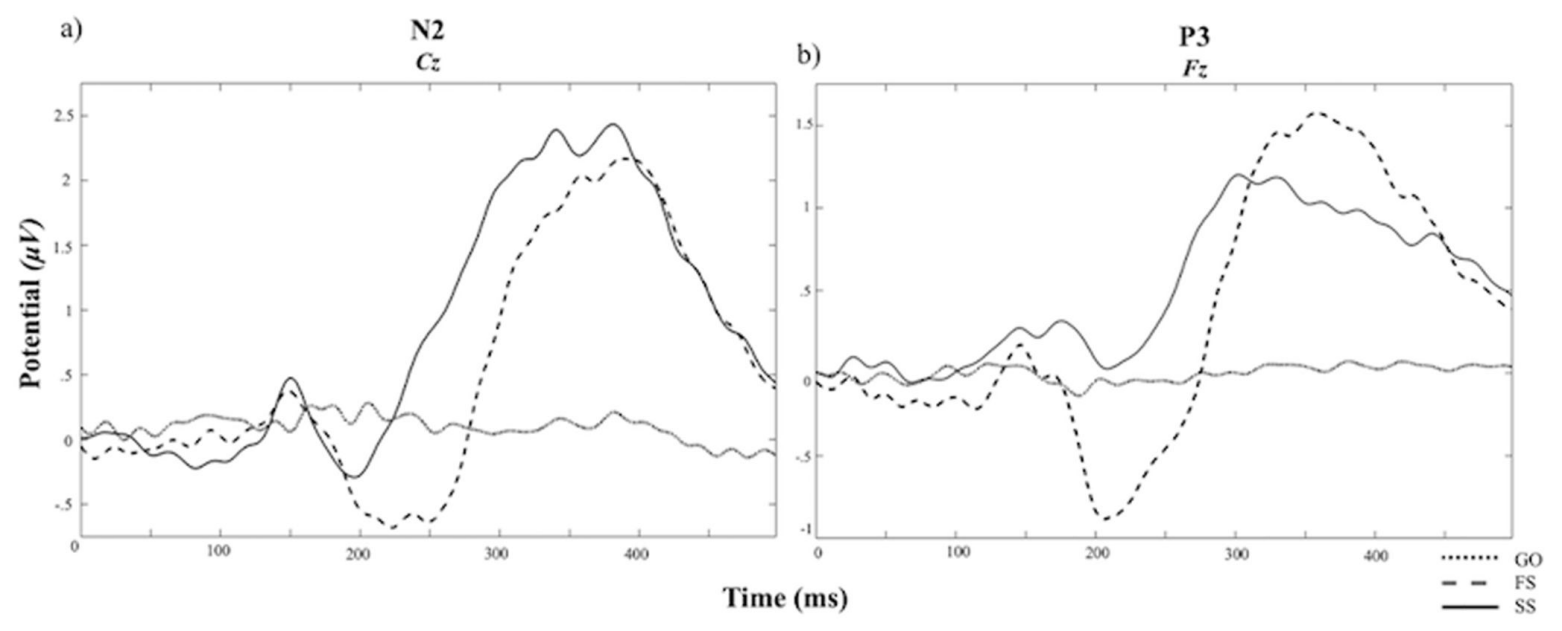
Grand Average of The N2 from the Cz Electrode and The P3 from the Fz Electrode Abbreviation: μV, Microvolts. Ms, Millisecond. FS, = Failed stop trials during the SST (i.e., red arrow presented at 0ms and participants incorrectly responded). SS, = Successful stop trials during the SST (red arrow presented at 0ms and participants correctly withheld a response). GO, = Go trials during the SST (white arrow presented at 0ms and participants responded).

**Figure 3:**
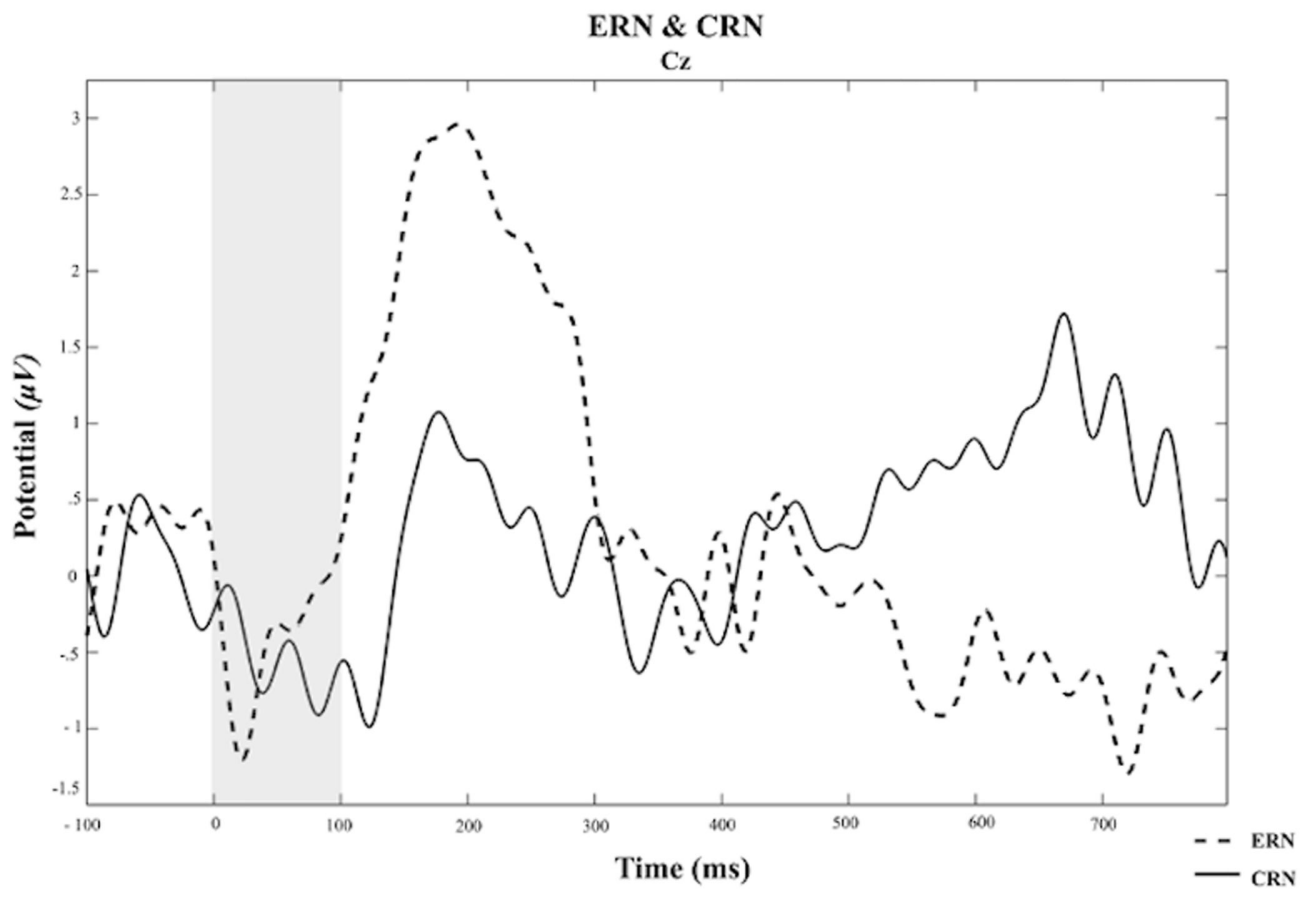
Grand Average of The ERN and CRN from the Cz Electrode Abbreviation: μV, Microvolts. Ms, Millisecond. ERN, Error Related Negativity. CRN, Correct Related Negativity. Note: Response (‘correct’ response - withholding a response to the ‘stop signal’, ‘incorrect’ response - responding to the ‘stop signal’) occurred at 0ms, shaded area corresponds to the 0-100ms period in which negativity was calculated. ERN and CRN index negativity after incorrect and correct responses, respectively.

Consistent with the prior research using the same version of the SST^39^, latency of the P3 onset was significantly earlier during successful as compared to failed stop trials (*t* (54) = 7.68, *p* < .001, 95% CI [77.26, 131.84]) (Figure 2, b). There were no significant differences between successful and failed stop trials in N2 or P3 amplitudes (*t* (71) = -.48, *p* = .63, *t* (54) = -0.26, *p* = .79, respectively). There were no significant differences in negativity between CRN and ERN (*f* (71) = .49, *p* = .63) (Figure 3).

### Inhibitory Control

#### N2 Amplitude

**Figure 4:**
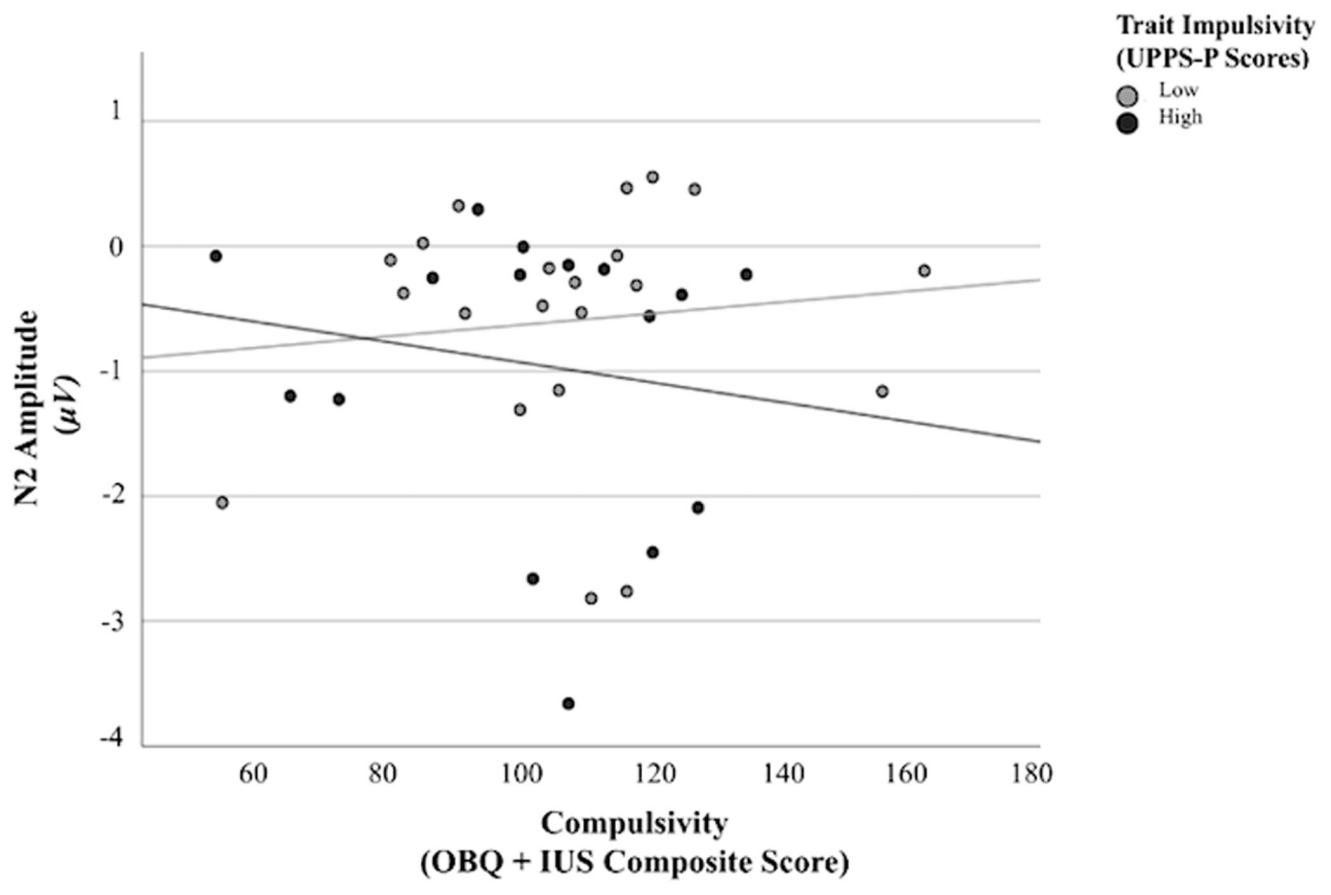
N2 Amplitude During Failed Stop Trials as a Function of Compulsivity Across Low and High Impulsivity Abbreviation: μV, Microvolts. UPPS-P, Urgency, Premeditation (lack of), Perseverance (lack of), Sensation Seeking, Positive Urgency, Impulsive Behavior Scale. OBQ, Obsessional Beliefs Questionnaire. IUS, Intolerance of Uncertainty Scale

For failed stop trials, the combination of impulsivity, compulsivity and their interaction accounted for 12% of variance in N2 amplitude (*R*^2^ = .12). The interaction between impulsivity and compulsivity was associated with significant variation in N2 amplitude (*F* (3, 33) = 1.48, *p* < .05, 95% CI [-.013,.001], *B* = .007 (*SE* = .003)). No significant correlation was found between compulsivity and N2 amplitude for high and low impulsivity groups (*r* = -.16, *p* = .56; *r* = .11, *p* = .63, respectively). Visual depiction of scatterplot data (Figure 4) indicated that the significant interaction effect was driven by a positive relationship between compulsivity and N2 amplitude amongst individuals with low impulsivity, and a negative relationship between compulsivity and N2 amplitude amongst individuals with high impulsivity.

For successful stop trials, impulsivity, compulsivity, and their interaction were not associated with significant variation in N2 amplitude (*F* (3, 33) = 1.11 *,p =* .36).

#### P3 Onset

**Figure 5:**
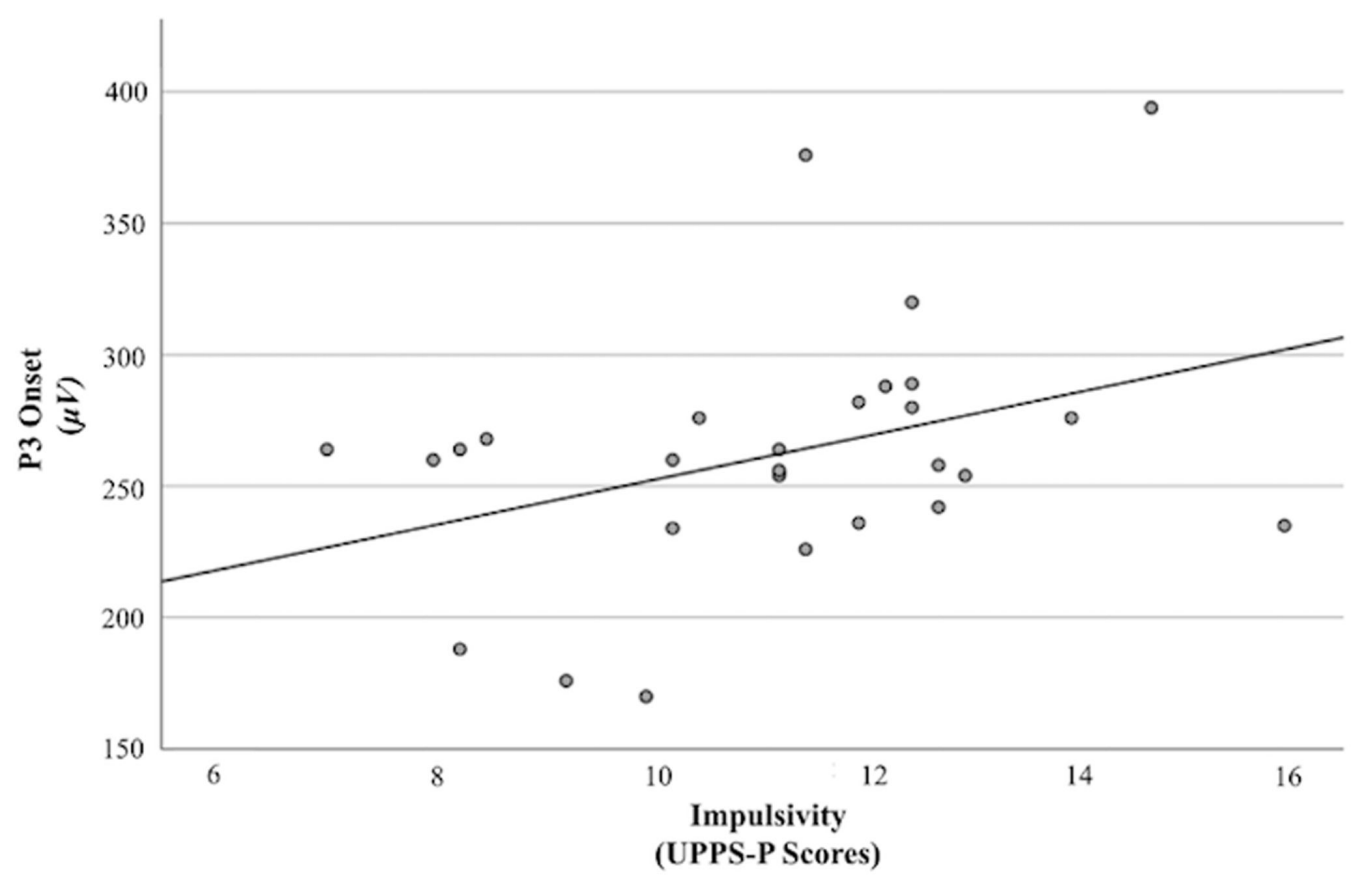
P3 Onset During Successful Stop Trials as a Function of Impulsivity Abbreviation: μV, Microvolts. UPPS-P, Urgency, Premeditation (lack of), Perseverance (lack of), Sensation Seeking, Positive Urgency, Impulsive Behavior Scale.

For successful stop trials, the combination of impulsivity, compulsivity and their interaction accounted for 23% of variance in P3 onset (*R*^2^ = .23). Impulsivity was associated with a trend level relationship with greater P3 onset latency (*F* (3, 24) = 2.33, *p* = .099), such that for every unit increase in impulsivity, P3 onset latency increased by 6.84 units (*B* = 6.84 *(SE =* 5.81) (Figure 5). Compulsivity and the interaction between impulsivity and compulsivity were not associated with significant variation in P3 onset latency (*p*’s < .28)

For failed stop trials, impulsivity, compulsivity, and their interaction were not associated with significant variation in P3 onset latency (*F* (3, 26) = 1.11, *p* = .36).

#### P3 Amplitude

**Figure 6:**
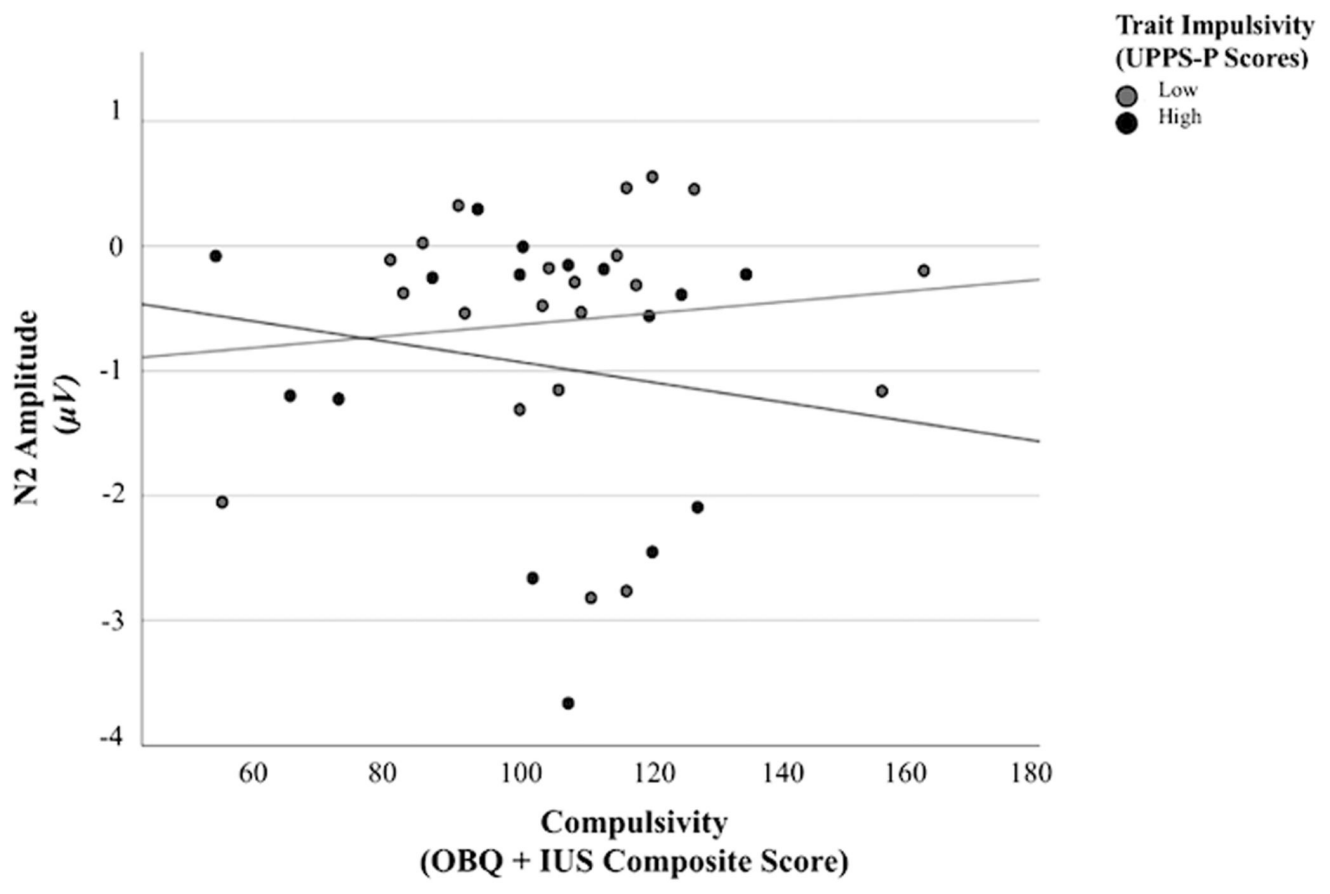
P3 Amplitude During Failed Stop Trials as a Function of Compulsivity Across Low and High Impulsivity Abbreviation: μV, Microvolts. UPPS-P, Urgency, Premeditation (lack of), Perseverance (lack of), Sensation Seeking, Positive Urgency, Impulsive Behavior Scale. OBQ, Obsessional Beliefs Questionnaire. IUS, Intolerance of Uncertainty Scale. Note. Significant correlation between compulsivity and P3 amplitude in low impulsivity (*r =* -.544, *p* < .05). Microvolts (μV). UPPS-P = Urgency, Premeditation (lack of), Perseverance (lack of), Sensation Seeking, Positive Urgency, Impulsive Behavior Scale. OBQ = Obsessional Beliefs Questionnaire. IUS = Intolerance of Uncertainty Scale.

For failed stop trials, the combination of impulsivity, compulsivity and their interaction accounted for 20% of variance in P3 amplitude (*R*^2^ = .20). The interaction between impulsivity and compulsivity was associated with significant variation in P3 amplitude (*F* (3, 24) = 2.03, *p* < .05, 95% CI [-.002, .045], *B* = .019 (*SE* = .01)). No significant correlation was found between compulsivity and P3 amplitude in high impulsivity (*r* = -.27, *p* = .40), however there was a significant correlation between compulsivity and P3 amplitude in low impulsivity (*r* = -.54, *p* < .05). Visual depiction of scatterplot data (Figure 6) indicated that the significant interaction effect was driven by a positive relationship between P3 amplitude and compulsivity amongst individuals with low levels of impulsivity.

For successful stop trials, impulsivity, compulsivity, and their interaction were not associated with significant variation in P3 Amplitude (*F* (3, 24) = .50, *p =* .69).

### Self-Monitoring

#### ERN

Impulsivity, compulsivity, and their interaction were not associated with significant variation in ERN (*F* (3, 33) = .63, *p =* .60).

#### CRN

**Figure 7:**
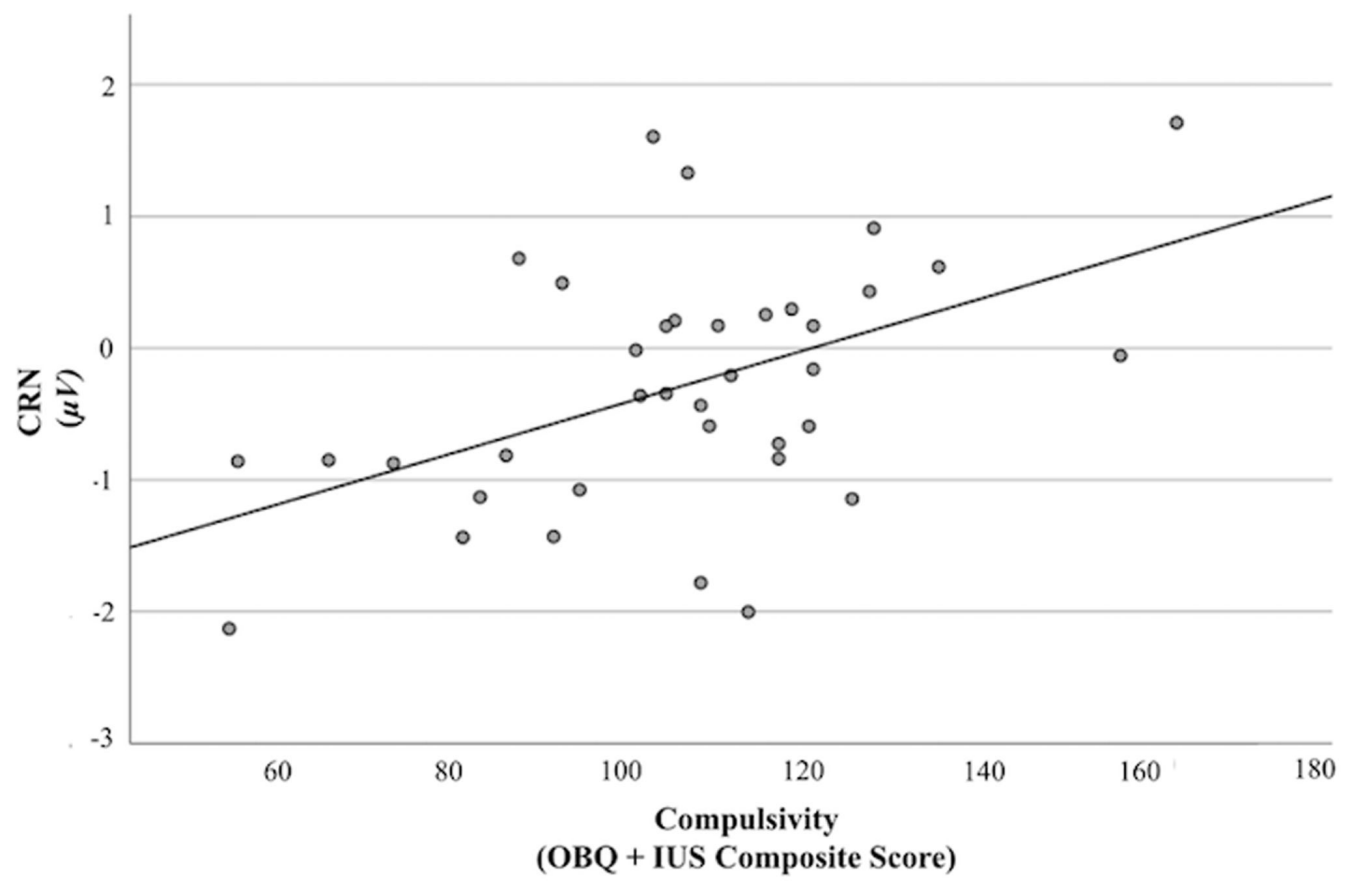
CRN as a Function of Compulsivity Abbreviation: μV, Microvolts. OBQ, Obsessional Beliefs Questionnaire. IUS, Intolerance of Uncertainty Scale. *Note.* Trait compulsivity was associated with larger CRN (*p <* .05). *Microvolts (nV).* OBQ = Obsessional Beliefs Questionnaire. IUS = Intolerance of Uncertainty Scale.

The combination of impulsivity, compulsivity and their interaction accounted for 37% of variance in CRN (*R*^2^ = .37). Compulsivity was associated with larger CRN when controlling for age (*F* (4, 32) = 4.76, *p <* .05, 95% CI [.01, .02]), such that for every unit increase in compulsivity, CRN increased by .02 units (*B* = .02 (*SE =* .01) (Figure 7). Impulsivity and the interaction between impulsivity and compulsivity were not associated with significant variation in CRN (*p* = .65, *p =* 54)

## Discussion

This was the first study to investigate whether varying traits of impulsivity and compulsivity across OCDS could be differentiated by inhibitory control and self-monitoring, as indexed by ERPs. The results indicated that ERPs indexing inhibitory control and self-monitoring did differentiate between impulsive and compulsive phenotypes in OCDS and can contribute to our understanding of the neurocognitive drivers of these traits and symptoms. In particular, when impulsivity was high and compulsivity low, failed inhibitory control was associated with enhanced N2 amplitude, reflecting high conflicts when trying to stop a behaviour. Further, as compulsivity increased, symptom severity also increased and CRN decreased, indicating reduced monitoring of successful inhibitory control. Taken together, two distinct phenotypes, i) high impulsivity/compulsivity and ii) high compulsivity, were identified and their unique neurocognitive profiles were characterised, i) poor inhibitory control (enhanced N2 amplitude) and ii) impaired self-monitoring (reduced CRN), respectively.

The finding that high impulsivity and compulsivity were associated with enhanced N2 amplitude is consistent with recent evidence^64^ implicating a disrupted N2 amplitude as a marker of an OCD-specific frontal cortical dysfunction that subserves impaired inhibitory control. The current findings extend this evidence by identifying an enhanced N2 amplitude in individuals high in impulsivity and compulsivity at the milder earlier stages in OCDS progression, as most people in this study do not yet meet an OCD diagnosis. Source localisation techniques have associated the N2 amplitude with prefrontal networks ^65,66^, including the IFG^67^, which drive inhibitory control^29,68,69^. In line with this, evidence has positioned N2 amplitude to reflect the conflict between the need to stop and the response urge^38,70^. Thus, people high in both impulsivity and compulsivity may experience higher conflicts, i.e., enhanced N2 amplitude, between recognising the need to stop unhelpful compulsive behaviours (e.g., awareness of excessive handwashing) and succumbing to urges (e.g., continuing to wash hands). These individuals, high in both impulsivity/compulsivity, show greater OCD severity^25^ and chronicity^24^, higher risk for developing clinically elevated impulse control and compulsive disorders ^71^, and poorer prognosis^26^. Thus, the identification of specific inhibitory control impairments, reflected by an enhanced N2 amplitude, in those with high impulsivity and compulsivity could form the basis for the early detection of people that meet this high-risk phenotype, and guide the development of early interventions that are tailored to specifically modulate inhibitory dyscontrol.

Impairments across self-monitoring were associated with high compulsivity. The findings that indicated this were i) a greater monitoring of failed stopping (enhanced ERN) across all individuals with OCDS, as compared to healthy norms ii) high compulsivity, independent of impulsivity, associated with lower monitoring of correct performance (lower CRN), and iii) high compulsivity, but low impulsivity, associated with poorer evaluation of failed performance (reduced P3 amplitude during failed stopping)^58,60,72–75^. of note, the P3 amplitude specifically during failed stopping on the SST^58,60,72^ (not sucessful stopping) has not shown robust and consistent evidence for indexing either inhibitory control or self-monitoring performance, and thereby CRN will be used as the stronger index of impaired self-monitoring. These findings are consistent with robust evidence that has implicated impaired self-monitoring in OCD via ERN/ERN findings^33,44^ and disruptions across anterior cingulate cortex (ACC) activity^76,77^. Most of the prior studies have been conducted on individuals diagnosed with OCD and have found error-related alternations^33,44^. This study provides further evidence of hypo-monitoring of correct performance and greater symptoms severity in highly compulsive people experiencing mild to moderate OCDS, which is earlier in symptom progression than those included in prior clinical studies. In highly compulsive individuals, hypo monitoring of correct performance, indexed by decreased CRN (e.g., difficulty recognising that hands are sufficiently clean), combined with hyper monitoring of errors indexed by enhanced ERN (e.g., excessively feeling that handwashing is incorrect), may reinforce compensatory, compulsive behaviours (e.g., inability to feel that handwashing is complete)^32,33,78^. This further neurocognitively characterises (impaired self-monitoring) specific phenotypes (high compulsivity) at the milder end of the OCDS severity continuum.

Consistent with literature^28,69,79^, high impulsivity was associated with a non-significant trend towards a delayed inhibitory control process, indexed by delayed P3 onset^39^. Taken together, those high in impulsivity may experience an additional, earlier slowing of the inhibitory control process. In other words, impulsive individuals may find it difficult to delay or stop behavioural urges, such as resisting compulsive handwashing, from the onset of the urge.

Neurocognitive phenotyping of impulsivity and compulsivity in OCDS could allow at-risk people to be identified earlier for specific interventions that target the drivers of symptoms. More specifically, most participants in the current study did not meet a diagnosis of OCD and were at the early stages of symptom progression (mild-moderate OCDS). Thus, the aforementioned findings collectively characterise the neurocognitive and mechanisms at the lower end of the dimensional spectrum of OCDS, where people are likely in the early ‘at-risk’ stages. This is the first step in enabling early detection of people that may be susceptible to OCD based on their neurocognitive and trait profile. Ultimately, this could enable the development of preventative or early treatments that specifically target the underlying mechanisms of a person's symptoms. These tailored early interventions could, for instance, utilise the knowledge from neurocognitive phenotyping to specifically modulate impairments in self-monitoring in people that fit the high compulsivity phenotype, or modulate impairments across inhibitory control in people that fit the high impulsivity/compulsivity phenotype. This evidence directly informs the ‘phenotype-to-treatment’ approach proposed by Yiicel, Lee, Fontenelle^27^ and is aligned with frameworks proposing the use of interventions to treat underlying drivers of symptoms, such as neurocognitive impairments, as opposed to categorical disorders ^24,29,36^. Whilst investigations into interventions for targeting inhibitory control are growing^80^, these are not yet established, and there has been minimal focus on remediating disordered self-monitoring. Thus, future investigations of interventions for subclinical OCDS will benefit from targeting the associated neurocognitive mechanisms (i.e., inhibitory control and self-monitoring) across impulsive and compulsive phenotypes to effectively remediate the underlying drivers of symptoms.

The current findings should be considered in light of a number of limitations. Firstly, the focus on mild to moderate severities means that the findings cannot be generalised to more severe OCD presentations. This is important to address in future research as these individuals are likely to be most in demand for earlier assessment and prevention, or more rigorous treatment. The sample is also disproportionately biased towards women. In order to be able to generalise the findings to broader populations, this study should be replicated in larger and more gender balanced samples. Additionally, the lack of a healthy control group as a comparator makes it difficult to disentangle whether the neurocognitive phenotypes were OCDS-specific and not reflective of normative traits within healthy populations. Finally, the results reported from the split group follow up approach, particularly the trend-level result for P3 onset, were based on small sample sizes. Thus, the study would benefit from replication with greater sample sizes.

Another important consideration is that, similar to findings by Wessel, Aron^39^, P3 onset was a more robust marker of successful inhibitory control than N2 and P3 amplitudes. More specifically, it was the only ERP that differentiated between successful versus unsuccessful inhibitory control during the SST. It is possible that this was a result of the same task used in this study and Wessel, Aron^39^, as ERPs are sensitive to task metrics ^37,81,82^. Additionally, given that the P3 amplitude is proposed to be an indicator of the magnitude of the (successful or failed) inhibition response, it may have captured the motor inhibition response (clicking the key or withholding) regardless of stopping success ^83,84^. Further, the P3 amplitude, particularly during failed stopping, lacks specificity and has been proposed to also influence attentional processes^37,85,86^. Thus, the evidence currently implicates the P3 onset as a more consistent indicator of successful inhibitory control, as compared to the N2 and P3 amplitudes.

## Conclusion

The current study found distinct inhibitory control and self-monitoring profiles, indexed by ERPs, across impulsive and compulsive phenotypes in individuals with mild to moderate OCDS. Firstly, those high in both impulsivity and compulsivity showed greater conflicts when stopping a response during failed inhibitory control, indexed by enhanced N2 amplitude. Secondly, those high in only compulsivity showed impairments in self-monitoring that had not yet been documented in the literature, reflect by a reduced CRN, and showed worsened symptom severity. These findings support the use of ERPs for identifying neurocognitive phenotypes across OCDS. This could inform a dimensional approach to characterising OCDS and its underlying mechanisms, that extends beyond binary diagnostic labels. Ultimately, mechanistic-based dimensional frameworks can enable earlier detection, and thereby, allow for early interventions that target the underlying neurocognitive mechanisms of distinct phenotypes across OCDS.

## Figures and Tables

**Table 1:** Participant Characteristics Across Variables and Normative Data for SSRT, P3 Onset, ERN/CRN and Well-Being

Variable	Normative Comparison	Mean	SD
Impulsivity	N/A	11.03	2.12
Compulsivity	N/A	106.72	21.61
YBOCS	Mild=1-15 Moderate =16-23	15-75	5.12
WEMWBS	Healthy Young Adults Median: 51, 95% CI 50 – 53	Median: Mild: 28.5 Moderate: 37	95% CI: Mild: 26.61 – 35.38 Moderate: 30.76 – 40.33
	People Accessing Secondary Care Mental Health Service Mean (SD): = 34.9 (13.8)	Mean: Mild: 31 Moderate: 35.55	SD: Mild: 8.81 Moderate: 10.79
SSRT (*ms*) N2 Amplitude (*μV*)	208.6 (SD = 75.1)	215.72	31.35
Failed Stop Trials	N/A	-.68	1.09
Successful Stop Trials	N/A	-.52	.99
P3 Onset Latency (*ms*)			
Failed Stop Trials	259.9 (SE = 6)	303.58	37.95
Successful Stop Trials	225.2 (SE = 4.5)	257.50	43.16
P3 Amplitude (*μV*)			
Failed Stop Trials	N/A	1.90	2.09
Successful Stop Trials	N/A	2.22	2.29
ERN (*μV*)	3.27 (SD = 6.56)	-.21	1.91
CRN (*μV*)	9.02 (SD = 5.29)	-32	.75

Abbreviation: M, Means. SD, Standard Deviations. SE, Standard Error. Ms, Milliseconds. μV, Microvolts. Note. There are not reliable normative data for N2 and P3 amplitudes during the SST.
